# Impact of Polyrhodanine Manganese Ferrite Binary Nanohybrids (PRHD@MnFe_2_O_4_) on Osteoblasts and Osteoclasts Activities—A Key Factor in Osteoporosis Treatment

**DOI:** 10.3390/ma15113990

**Published:** 2022-06-03

**Authors:** Krzysztof Marycz, Anna Kowalczuk, Eliza Turlej, Emilia Zachanowicz, Anna Tomaszewska, Magdalena Kulpa-Greszta, Martyna Kępska, Zofia Steczkiewicz, Robert Pązik

**Affiliations:** 1 Department of Experimental Biology, Wroclaw University of Environmental and Life Science, Norwida 27, 50-375 Wroclaw, Poland; eliza.turlej@upwr.edu.pl (E.T.); martyna.kepska@upwr.edu.pl (M.K.); zofia.steczkiewicz@upwr.edu.pl (Z.S.); 2 National Medicines Institute (NIL), Chelmska 30/34, 00-725 Warszawa, Poland; a.kowalczuk@nil.gov.pl; 3 Polymer Engineering and Technology Division, Wroclaw University of Technology, Wyspianskiego 27, 50-370 Wroclaw, Poland; emilia.zachanowicz@pwr.edu.pl; 4 Department of Biotechnology, Institute of Biology and Biotechnology, College of Natural Sciences, University of Rzeszow, Pigonia 1, 35-310 Rzeszow, Poland; atomaszewska@ur.edu.pl (A.T.); mkulpa@ur.edu.pl (M.K.-G.); rpazik@ur.edu.pl (R.P.); 5 Faculty of Chemistry, Rzeszow University of Technology, Powstancow Warszawy 12, 35-959 Rzeszow, Poland

**Keywords:** osteoporosis, osteoblasts/osteoclasts, polyrhodanine manganese ferrite, binary nanohybrids, apoptosis, inflammation, bone remodeling markers

## Abstract

Osteoporosis is characterized by the reduction of bone mineral density and the weakness of the bone strength leading to fractures. Searching for new compounds that stimulate bone activity and their ability to reconstruct seems to be a promising tool in osteoporosis treatment. Here, we performed analyses comparing the impact of polyrhodanine (PRHD) and its derivatives on the viability (anti-proliferative tests), morphology and mitochondrial network (confocal microscopy) towards pre-osteoblasts (MC3T3-E1 cell line) and osteoclasts (4B12 cell line). Moreover, we assessed the expression of genes associated with the apoptosis, inflammation and osteogenic differentiation by qPCR technique. Our results clearly demonstrated that PRHD and its modification at ratio 10/90 significantly improves the pre-osteoblast’s proliferative abilities, while reducing osteoclast function. The observed effects were strongly correlated with the cytoskeleton and mitochondrial network development and arrangement. Additionally, the expression profile of genes revealed enhanced apoptosis of osteoclasts in the case of PRHD and its modification at ratio 10/90. Moreover, in this case we also observed strong anti-inflammatory properties demonstrated by decreased expression of *Il1b, Tnfa* and *Tgfb* in pre-osteoblasts and osteoclasts. On the other hand, enhanced expression of the markers associated with bone remodeling, namely, osteopontin (OPN), osteocalcin (OCL) and alkaline phosphatase (ALP), seem to confirm the role of PRHD@MnFe_2_O_4_ in the promotion of differentiation of pre-osteoblasts through the *ALP-OPN-OCL* axis. Based on these observations, PRHD@MnFe_2_O_4_ could be a potential agent in osteoporosis treatment in future, however, further studies are still required.

## 1. Introduction

Nowadays, osteoporosis has become one of the most common bone disorders in societies all over the world due to progressive aging. It is characterized by reduced bone mass and mechanical properties that predispose to bone fracture. Osteoporosis is most common in women and elderly patients, who are more prone to osteoporosis incidents due to their age and senescence related disorders [1]. Although it was thought thus far, that osteoporosis becomes a syndrome characterized by back pain, osteopenia and vertebral fractures, it is now classified as a primary disorder of the skeleton related to profound metabolic changes not only in bone, but also related to changes in whole body homeostasis. Therefore, osteoporosis has not only become a serious risk factor for bone fracture, but also as reported recently for hypertension and stroke [2]. It is estimated by the World Health Organization (WHO), that only in the UK and USA, around 400–800 patients per 100,000 women will have bone fractures incidents. Moreover, the WHO estimates that more than 5 million American men are affected by osteoporosis, based on recorded bone fracture incidents (humerus/tibial, hip or wrist fractures) or significantly low bone mineral density (BMD). These alarming data indicate the necessity for searching for effective therapies for osteoporosis related bone fractures [3].

One of the critical components of osteoporosis development is estrogen deficiency and progressive aging of bone cells that leads to bone microstructure deterioration and finally to bone lose. Impaired mechanism could maintain bone homeostasis leading to reduced bone mineral density and in consequence to bone fractures [4]. Previously, it was shown that the loss of balance between bone forming (osteoblasts) and resorbing cells (osteoclasts) was a main factor initiating osteoporosis development. In the course of the bone formation process, osteoblasts, that differentiate from bone marrow-derived mesenchymal stem progenitor cells (BMSCs), deposit collagen, morphogenetic protein, alkaline phosphatase (ALP) and finally minerals. Once the osteoblasts have completed their function, they become flattened and differentiated into mature osteocytes and undergo apoptosis. In turn, osteoclasts, by using a wide range of enzymes and hydrogen ions being attached to the bone surface, initiated the bone resorption process through breakdown of bone matrix. The bone matrix is composed of both inorganic (hydroxyapatite) and organic phases (collagen, proteoglycans and glycoproteins), both degraded by osteoclasts. These fundamental processes of bone formation, mediated by osteoblasts and osteoclasts, are seriously impaired in patients with osteoporosis by osteoclast overactivity, that in turn leads to the phenomenon in which osteoporotic patients lose more bone matrix than the osteoblasts are able to produce. This mechanism is believed to be critical for understanding the mechanisms that could potentially rescue osteoblasts from these unfavorable conditions [5].

The deteriorated bone remodeling is strongly related to enhanced apoptosis, that is triggered by different pathways including ligand activation via death receptors that belongs to the tumor necrosis factor receptor superfamily (TNFRSF), TNF-related apoptosis-inducing ligand (TRAIL) R-1 and R-2 and interleukin-1 (IL-1) and 6 (IL-6). It is considered, that TNFα (tumor-necrosis factor α) and IL-1 or IL-6 related apoptosis might become a therapeutic target, since osteoporosis exhibits elevated systemic of pro-inflammatory cytokines expression [6]. Moreover, the differentiation process of the pre-osteoblasts is widely recognized as an important mechanism that participates in bone formation. This process is mediated by the ALP, dentin matrix acidic phosphoprotein 1 (DMP1), osteocalcin (OC)—also known as bone gamma-carboxyglutamic acid-containing protein (BGLAP)—and osteopontin (OPN). ALP is specific for bone formation and becomes a diagnostic marker in the course of osteoporotic patients, only when no bile duct or liver disease are diagnosed. DMP1 has recently been demonstrated as an extracellular matrix (ECM) protein enriched in bone tissue and terminal cells, that promotes osteoblasts and osteocytes maturation and finally leads to mineralization. OPN and OC are known as specific products of differentiated osteoblasts and are strongly involved in maintaining cell survival and proliferative activity, while modulating the mineralization process and extracellular matrix formation. Therefore, targeting the *ALP-OPN-OCN* axis in osteoblasts to enhance the bone formation process seems to be a fully reasonable strategy for improved osteoporosis related bone fracture regeneration [7].

Recently, polyrhodanine manganese ferrite binary nanohybrids (PRHD@MnFe_2_O_4_) have been identified as an attractive source of superparamagnetic iron nanoparticles for potential biomedical application [8]. Due to their unique properties, superparamagnetic iron oxide nanoparticles (MNPs) have been proposed as a good candidate not only for bioimaging field of medicine, but also as an active agent that could promotes adipose stem progenitor cells’ (ASCs) proliferative activity [9]. Since then, observation has been made that MnFe_2_O_4_ might modulate osteoblast/osteoclast activity, and therefore becomes interesting agent for modulation of the bone remodeling process. To protect the cells from the unpredictable effect of MNPs (Fenton’s reactions), MnFe_2_O_4_ nanoparticles might be covered by various polymers including polyrhodanine. For that reason, we fabricated polyrhodanine manganese ferrite binary nanohybrids (MnFe_2_O_4_) and we investigated their effect on osteoblast precursor and osteoclasts activity.

We found that PRHD@MnFe_2_O_4_ reduced inflammation, apoptosis and promoted differentiation potential of osteoblast progenitors, while inhibiting osteoclast activity via the *ALP-OPN-OCN* axis.

## 2. Materials and Methods

### 2.1. Fabrication of the PRHD@MnFe_2_O_4_ Binary Nanohybrids

Protocols of the MnFe_2_O_4_ nanoparticles and PRHD@MnFe_2_O_4_ composites synthesis were described by us in detail elsewhere [8,10]. Metal acetylacetonate complexes were weighted under a protective atmosphere of N_2_ (acrylic glovebox P10R250T2, GS Glove Box Systemtechnik GmbH, Malsch, Germany). Namely, 0.6329 g (2.5 mmol) of Mn(acac)_2_ (99.9%, Alfa Aesar, Kandel, Germany) and 1.7658 g (5 mmol) of Fe(acac)_3_ (99.99%, Alfa Aesar, Kandel, Germany) were dissolved in 70 mL of acetophenone (99%, Sigma Aldrich, Steinheim am Albuch, Germany) and directly transferred into the microwave reactor (Ertec, Wroclaw, Poland). The process was conducted for 60 min at 200 °C and 15 atm. After cooling down the reaction mixture, stock MnFe_2_O_4_ nanoparticles were separated via fast centrifugation, washed several times with portions of ethanol (96%, Avantor, Gliwice, Poland) and finally resuspended in de-ionized water. The resulting MnFe_2_O_4_ stock suspension was further used to prepare PRHD@MnFe_2_O_4_ composites through the oxidation polymerization process. Briefly, in order to prepare the PRHD@MnFe_2_O_4_ 10%@90% (1:10 sample), 5 mg of rhodanine monomer (C_3_H_3_NOS_2_ 99%, Alfa Aesar, Kandel, Germany) was dissolved in water at 70 °C under mechanical stirring together with 50 mg MnFe_2_O_4_ particles. The total volume was 100 mL. The polymerization process was initiated by the addition of FeCl_3_ (96%, Avantor, Gliwice, Poland) and left for 24 h at room temperature while maintaining stirring. PRHD@MnFe_2_O_4_ hybrids were washed three times using water and dried under vacuum at 40 °C for 24 h.

The structure of the stock MnFe_2_O_4_ and PRHD@MnFe_2_O_4_ hybrids was confirmed by X-ray powder diffraction technique (XRD) using a D8 Advance diffractometer (Bruker, Camarillo, CA, USA) equipped with Cu lamp by measurement of diffraction patterns in a 10–60° 2Θ range and comparison with reference standards taken from the ICDD database. The particle size and morphology of stock nanoparticles were determined through analysis of the transmission electron microscopy images collected with a CM-20 Super Twin microscope (Philips, Eindhoven, The Netherlands) operating at 200 kV. The standard procedure of sample preparation was adopted by placing a small droplet of nanoparticle suspension on a copper grid with a perforated carbon layer and slow evaporation at room temperature. Since the imaging of the polymer containing samples under high voltage is strongly problematic (irreversible sample damage, melting and decomposition), a Helios Nanolab 660 scanning electron microscope (Thermo Fischer Scientific, Waltham, MA, USA), working at a low voltage (2 kV), was employed to scan the hybrid materials. In this case, several droplets of a water suspension of PRHD@MnFe_2_O_4_ composite were deposited onto carbon tape and slowly dried (24 h at 25 °C). The presence of the PRHD was studied by Fourier transform infrared spectroscopy (FTIR) using a Nicolet iZ10 spectrometer (Thermo Fischer Scientific, Waltham, MA, USA) and attenuated total reflection (ATR) accessory (diamond crystal). The spectra were collected within 4000–500 cm^−1^ spectral range without any special pretreatment of the sample. To obtain information regarding the amount of outer polymer layer, thermogravimetric analysis (TGA) was performed using a TA-300 system (Mettler Toledo, Allison Park, PA, USA) in the temperature range of 25–650 °C and heating rate of 10 °C/min. Before measurements, apparatus was validated by using a reference calcium oxalate sample.

### 2.2. Cell Lines and Culture

In this study, we used two mouse derived cell lines: pre-osteoblast mouse cell line MC3T3-E1 subclone 4 purchased from the American Type Culture Collection (ATCC) and the mouse osteoclasts precursor cell line 4B12 kindly provided by-Shigeru Amano from the Department of Oral Biology and Tissue Engineering, Meikai University School of Dentistry. The pre-osteoblast cell line was cultured in minimum essential medium alpha without ascorbic acid (MEM-α, Gibco, Waltham, MA, USA) and supplemented with 10% fetal bovine serum (FBS, Merck, KGaA, Darmstadt, Germany) and 1% penicillin-streptomycin (Merck KGaA, Darmstadt, Germany). The osteoclast cell line was cultured in MEM-α, 30% calvaria-derived stromal cell conditioned media (CSCM) and 10% FBS as described previously [11]. Cells were maintained during all the experiments at constant conditions: 37 °C in a 5% CO_2_ atmosphere.

### 2.3. Cell Viability Assay

Cells were treated with MnFe_2_O_4_, PRHD or with PRHD@MnFe_2_O_4_ at different ratios (10:90 or 40:60) at a concentration 90.8 μg/mL. The viability of cells was determined after 24, 48 and 72 h of culture using resazurin-based assay (TOX-8, Merck KGaA, Darmstadt, Germany) according to the protocol. The absorbance was measured using a 96-well microplate reader (Epoch; Biotek Instruments, Winnoski, VT, USA). Spectrophotometric measurements were taken at 600 nm and 690 nm as the reference lengths. Each experiment was performed at least three times.

### 2.4. Fluorescent Microscopy

The mitochondria, actin filaments and the nucleus of the tested cells were stained as described previously [12]. Mitochondria were stained using MitoRed dye (Life Technologies, Carslbad, CA, USA), F-actin filaments using Phalloidin-Atto 488 staining (Life Technologies, Carslbad, CA, USA) and cell nuclei with 4′,6-diamidino-2-phenylindole DAPI (Life Technologies, Carslbad, CA, USA).

Briefly, the cells were incubated for 30 min with MitoRed solution (1:1000) at 37 °C and then fixed with 4% PFA (POCh Gliwice, Poland). According to the protocol, cells were stained with phalloidin for 45 min at RT and then with DAPI. Visualization was made by a confocal microscope (Leica TCS SPE, Leica Microsystems, Wetzlar, Germany) at 0.5 µm steps up to a final depth of 25 µm. Images were captured at magnification 630× and were analyzed using Fiji New ImageJ with Colour Pixel Counter plugin version 1.52 developed by Wayne Rasband from NIH, USA. Each photograph was taken at least three times independently.

### 2.5. Analysis of mRNA Expression Profiles

Cells were seeded on the plastic plates at the density of 1 × 10^4^/well in the appropriate medium. The total RNA was isolated using the acid guanidinium thiocyanate-phenol-chloroform extraction method described by Chomczynski and Sacchi [13]. The obtained RNA was diluted in DEPC-treated water (Merck KGaA, Darmstadt, Germany) and the quantity and quality of RNA were then measured using a nanospectrophotometer (Epoch, Biotek Instruments, Winnoski, VT, USA). Digestion of genomic DNA and cDNA synthesis was performed using Takara PrimeScript RT Reagent Kit with gDNA Eraser (Perfect Real Time) (Takara, Bio Europe, Göteborg, Sweden) and using a T100 Thermal Cycler (Bio-Rad, Hercules, CA, USA). For a single reaction, 150 ng of RNA was used and the procedures were carried out according to the manufacturer’s protocol. The cDNA templates from each cell were amplified by the quantitative reverse transcription polymerase chain reaction using SensiFAST SYBR No-ROX Kit (Bioline, London, UK) in total volume of each reaction of 10 µL (for a single reaction-1 μL of cDNA and 500 nM of each primer, according to the protocol). The sequences of the specific primers (Merck KGaA, Darmstadt, Germany) used in qPCR are listed in Table 1. The qRT-PCR reactions were performed using a CFX Connect Real-Time PCR Detection System (CFX Connect Optics Module, Bio-Rad, Hercules, CA, USA) equipped with BioRad CFX Maestro software and the transcript levels were normalized to *Gapdh* as a control (house-keeping gene).

### 2.6. Statistical Analysis

Statistical analysis was performed using GraphPad Prism 5 software and the statistical significance was marked with an asterisk (*). *p* value less than 0.05 (*p* < 0.05) are marked with one asterisk (*), *p* value less than 0.01 (*p* < 0.01) with two asterisks (**) and the *p* values less than 0.001 (*p* < 0.001) with three (***).

## 3. Results

### 3.1. Physicochemical Properties of the PRHD@MnFe_2_O_4_ Binary Hybrids

**Figure 1:**
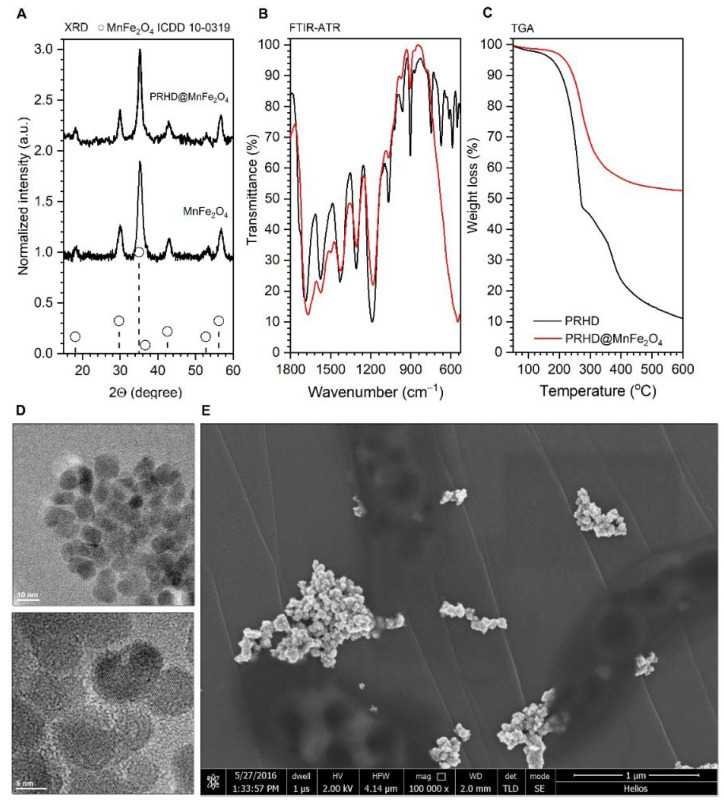
Representative results of the MnFe_2_O_4_ and PRHD@MnFe_2_O_4_ characterization arranged as follows: (**A**) XRD diffraction patterns of both materials; (**B**) FTIR-ATR spectra of the reference PRHD and composite; (**C**) TGA curves of the PRHD and PRHD@MnFe_2_O_4_; (**D**) TEM images of the stock MnFe_2_O_4_ nanoparticles as well as (**E**) SEM picture of the PRHD@MnFe_2_O_4_. Please refer [8].

Since the physicochemical properties of the hybrid materials were already a subject of our recent studies, the reader is strongly encouraged to familiarize themselves with the results presented earlier, where detailed characteristics can be found [8]. Here we would like to underline that both stocks of nanoparticles and composites are well defined since they are from the same synthetic batch in both articles. As one can note, XRD diffraction patterns (Figure 1A) were recorded for the stock MnFe_2_O_4_ nanoparticles as well as binary hybrid material PRHD@MnFe_2_O_4_, showing a good correspondence with the reference standard from the ICDD database card No. 10-0319 attributed to the MnFe_2_O_4_ spinel phase. Thus, the procedure of rhodanine polymerization does not affect the structural properties of the nanoparticles themselves. The FTIR-ATR characterization shows direct evidence of the PRHD presence on the MnFe_2_O_4_ surface since typical vibration modes ascribed to the polymeric component are clearly distinguishable together with the mode at around 590 cm^−1^ associated with the vibrations of the Fe-O bonds (Figure 1B). Moreover, TGA analysis (Figure 1C) supports the fact of the effective coverage of the MnFe_2_O_4_ with polymer layer showing distinct weight loss and effect of the MnFe_2_O_4_ on the thermal stability of the composite in contrast to pure polymer (extended discussion can be found in [8]. Figure 1D presents a typical transmission image of the stock nanoparticles fabricated using the microwave-driven technique. The particles are small and have irregular shapes, sometimes resembling spheres, which can point to a rather non-direction preferred particle growth with an average size close to 8.5 nm. MnFe_2_O_4_ is well crystallized since lattice fringes can be easily identified (Figure 1D bottom part). Imaging of the binary hybrid sample is trickier due to the possible sample damage due to the highly energetic electron beam used in TEM microscopy. Therefore, SEM was performed (Figure 1E) revealing the presence of larger objects with the size being slightly above 20 nm.

### 3.2. Cytotoxicity of the PRHD@MnFe_2_O_4_ at Different Concentration Ratios towards Osteoblasts and Osteoclasts

We analyzed the impact of the PRHD and its new manganese binary ferrite modifications on the kinetics of the proliferation of the pre-osteoclasts and pre-osteoblasts.

**Figure 2:**
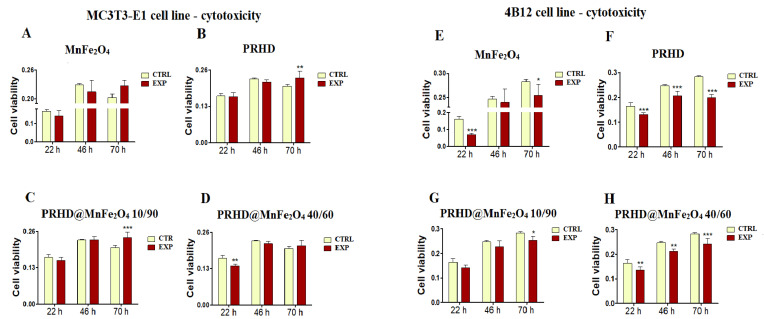
The impact of the polyrhodanine manganese ferrite binary nanohybrids and its modifications on the kinetics of the proliferation of MC3T3-E1 (pre-osteoblast) cell line (left side) and 4B12 (osteoclast) cell line (right side). CTRL: control, EXP: experimental probe The graphs represent: MnFe_2_O_4_ (**A**,**E**), PRHD (**B**,**F**), PRHD@MnFe_2_O_4_ 10/90 (**C**,**G**), PRHD@MnFe_2_O_4_ 40/60 (**D**,**H**). Significant differences are indicated by * *p* < 0.005; ** *p* < 0.001 and *** *p* < 0.0001 (in comparison to control). Each graph represents data from three independent experiments.

Firstly, we determined the effect of PRHD modified only by MnFe_2_O_4_ at different concentration ratios (40/60 vs. 10/90) in comparison to controls (unstimulated cells or stimulated only with PRDH alone). Interestingly, PRHD and its modification using MnFe_2_O_4_ in relative to 10/90 concentration clearly enhanced the viability of the osteoblasts only after 70 h of incubation (Figure 2B,C).

Comparing the impact of the new modifications towards viability of osteoclasts (4B12 cell line), we noticed that both MnFe_2_O_4_ and PRHD used alone decreased osteoclast viability independently of selected time point (Figure 2E,F).

In case of the various concentrations of PRHD and MnFe_2_O_4_, the strongest decrease of the viability of osteoclasts was observed after using polyrhodanine modified by MnFe_2_O_4_ at ratio 40 to 60, independent of time point (Figure 2H), although PRHD modified with MnFe_2_O_4_ at ratio 10/90 after 70 h also revealed significant impact on the decrease of the osteoclasts’ viability (Figure 2G).

### 3.3. The Impact of the PRHD@MnFe_2_O_4_ on the Morphology and Mitochondrial Status of Osteoblasts and Osteoclasts

The effect of the PRHD and its modification on the morphology of the MC3T3-E1 and 4B12 cell line was assessed by confocal microscopy. The impact of them on the fundamental cellular processes, such as morphogenesis, migration ability and cell division, was determined using F-actin staining, while the mitochondrial status was determined using MitoRed staining.

**Figure 3:**
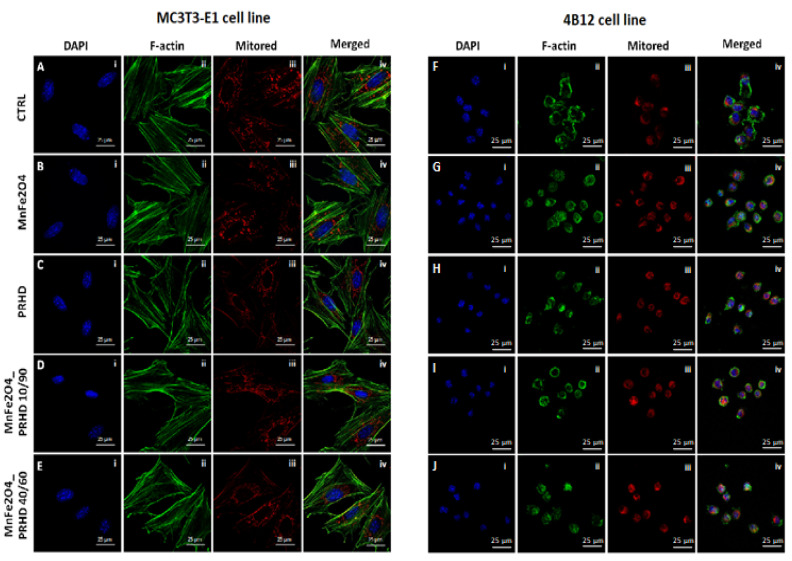
The impact of the polyrhodanine manganese ferrite binary nanohybrids and its modification on the morphology, cytoskeleton and mitochondria status on the MC3T3-E1 cell line (left side: **A**(**i**–**iv**)–**E**(**i**–**iv**)) or 4B12 cell line (right side: **F**(**i**–**iv**)–**J**(**i**–**iv**)). The photographs were captured at 630× magnification. Scale bar, 25 μm. Each staining was performed independently at least three times.

Our studies revealed that the cytoskeleton in osteoblasts is better developed after the PRHD@MnFe_2_O_4_ 40/60 in comparison to the control cells (Figure 3E(ii)). Mitochondrial staining revealed that, this modification caused the network arrangement (Figure 3E(iii)). In the case of osteoclasts, the best intensification of the cytoskeleton and mitochondrial staining is visible in the MnFe_2_O_4_-treated cells (Figure 3G(ii,iii)).

### 3.4. The Impact of the PRHD@MnFe_2_O_4_ on the Gene Expression in Osteoblasts and Osteoclasts

#### 3.4.1. Genes Associated with the Apoptosis

**Figure 4:**
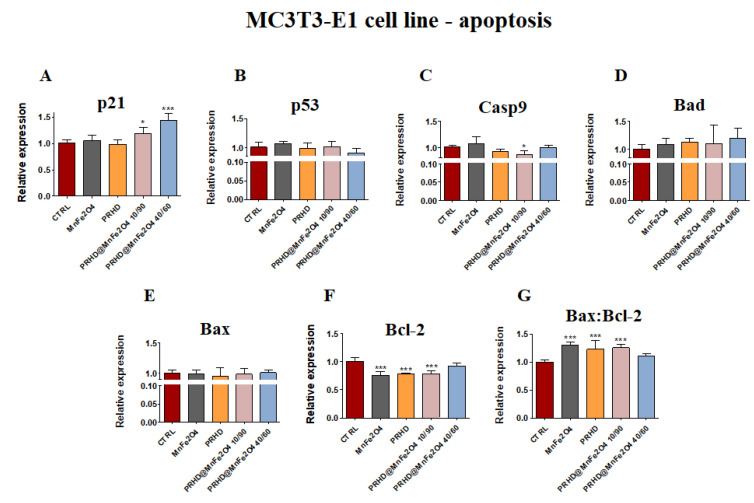
The impact of the polyrhodanine manganese ferrite binary nanohybrids and its modifications on the expression of *p21* (**A**), *p53* (**B**), *Casp9* (**C**), *Bad* (**D**), *Bax* (**E**), *Bcl-2* (**F**) and *Bax:Bcl-2* ratio (**G**) towards osteoblasts. Significant differences are indicated as follows: * *p* < 0.005, and *** *p* < 0.001. Each experiment was performed independently at least three times.

The analysis of gene expression associated with the apoptosis in the case of the osteoblasts showed that PRHD modifications using MnFe_2_O_4_ could influence the survival of cells. While osteoblasts treated with this modification independently of their ratio showed significant increase of the *p21* and the ratio of *Bax:Bcl-2* (Figure 4A,G), the significant decrease of the *Casp9* and *Bcl-2* was observed (Figure 4C,F). However, it is worth noting that this effect was visible only in the case of PRHD@MnFe_2_O_4_ at concentration 10/90.

**Figure 5:**
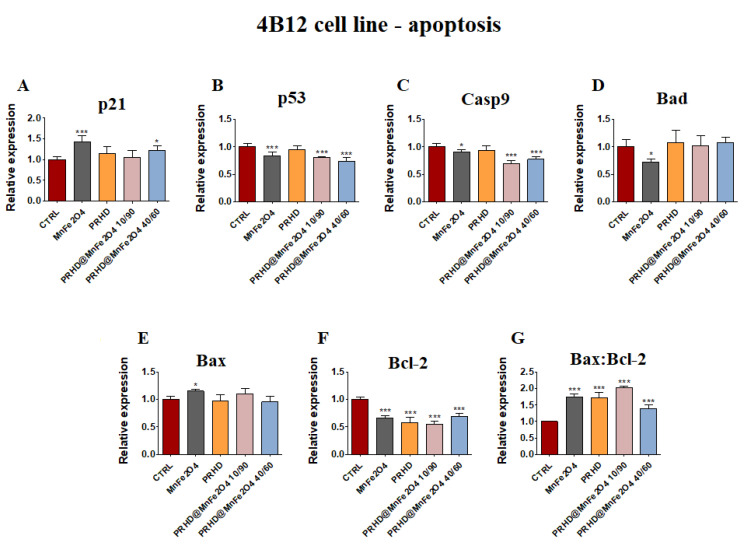
The impact of the polyrhodanine manganese ferrite binary nanohybrids and its modifications on the expression of *p21* (**A**), *p53* (**B**), *Casp9* (**C**), *Bad* (**D**), *Bax* (**E**), *Bcl-2* (**F**) and *Bax:Bcl-2* ratio (**G**) towards osteoclasts. Significant differences are indicated as follows: * *p* < 0.005, and *** *p* < 0.001. Each experiment was performed independently at least three times.

In the case of osteoclasts, it was noticed that the effect of the PRHD@MnFe_2_O_4_ independently of concentration caused the decrease of *p53, Casp9* and *Bcl-2* (Figure 5B,C,F), while the ratio of *Bax:Bcl-2* was significantly increased (Figure 5G). The effect of the MnFe_2_O_4_ was slightly changeable, and caused the increase of *p21* and *Bax,* while *p53, Casp9, Bad* and *Bcl-2* were decreased (Figure 5A–G).

#### 3.4.2. Genes Associated with the Inflammation

In parallel, the pro-inflammatory properties of newly prepared PRHD modifications were determined based on the mRNA expression of *Il1b, TNFα*, *iNOS*, *Il6* and *Tgfb1.*

**Figure 6:**
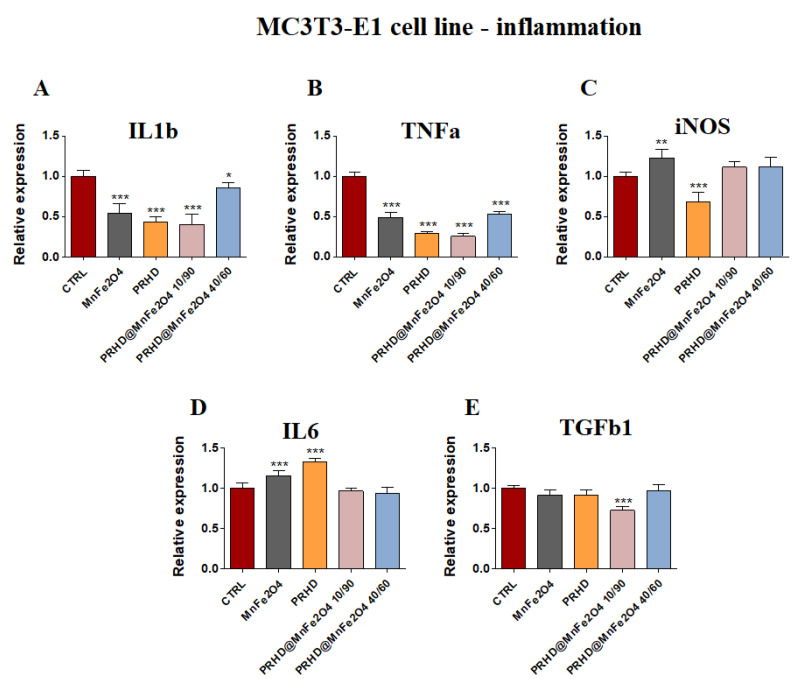
The impact of the polyrhodanine manganese ferrite binary nanohybrids and its modifications on the expression of genes associated with proinflammatory cytokines: *Il1b* (**A**), *Tnfa* (**B**), *iNOS* (**C**), *Il6* (**D**), *Tgfb* (**E**) towards osteoblasts. Significant differences are indicated as follows: * *p* < 0.005, ** *p* < 0.001 and *** *p* < 0.001. Each experiment was performed independently at least three times.

PRHD and its modifications were involved in the decreasing of expression of mRNA of *Il1β* and *Tnfα* (Figure 6A,B), although the expression of *Tgfb1* was decreased only in the case of using PRHD@MnFe_2_O_4_ at concentration 10/90 (Figure 6E). Interestingly, in both *iNOS* and in *Il6* cases, MnFe_2_O_4_ stimulated increasing mRNA expression, although PRHD alone indicated the opposite effect on their expression, increasing *Il6* and decreasing *iNOS* (Figure 6C,D).

**Figure 7:**
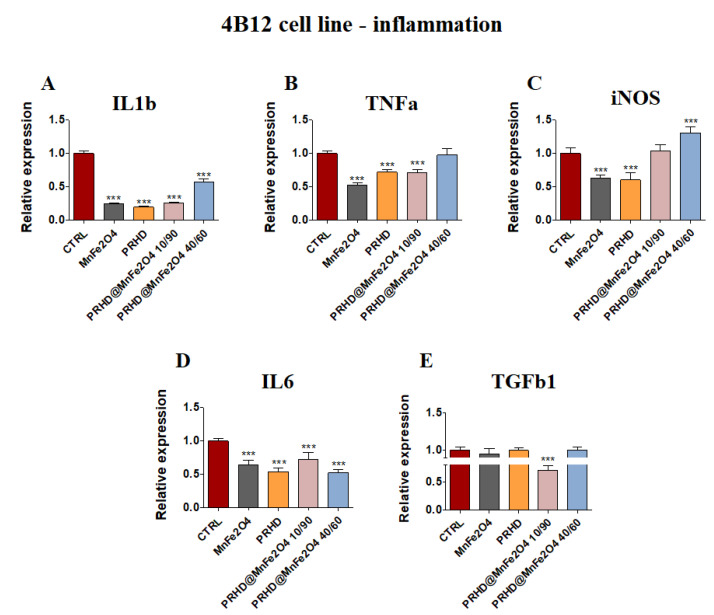
The impact of the polyrhodanine manganese ferrite binary nanohybrids and its modifications on the expression of genes associated with proinflammatory cytokines: *Il1b* (**A**), *Tnfa* (**B**), *iNOS* (**C**), *Il6* (**D**), *Tgfb* (**E**) towards osteoclasts. Significant differences are indicated as follows: *** *p* < 0.001. Each experiment was performed independently at least three times.

In parallel, it was observed that MnFe_2_O_4_ and PRHD alone as well as PRHD in combination with the manganese ferrite binary nanohybrids at concentration 10/90 clearly decreased the expression of mRNA of *Il1b, Tnfa* and *Il6* (Figure 7A,B,D) in the osteoclasts. Additionally, we observed the significant decrease of mRNA expression of *Tgfb1* (Figure 7E).

On the one hand, the combination of PRHD and MnFe_2_O_4_ at the 40/60 ratio decreased the expression of mRNA of *Il1b* and *Il6,* but on the other hand, increased the expression of *iNOS* (Figure 7A,D,C).

#### 3.4.3. Genes Associated with the Osteogenic Differentiation

**Figure 8:**
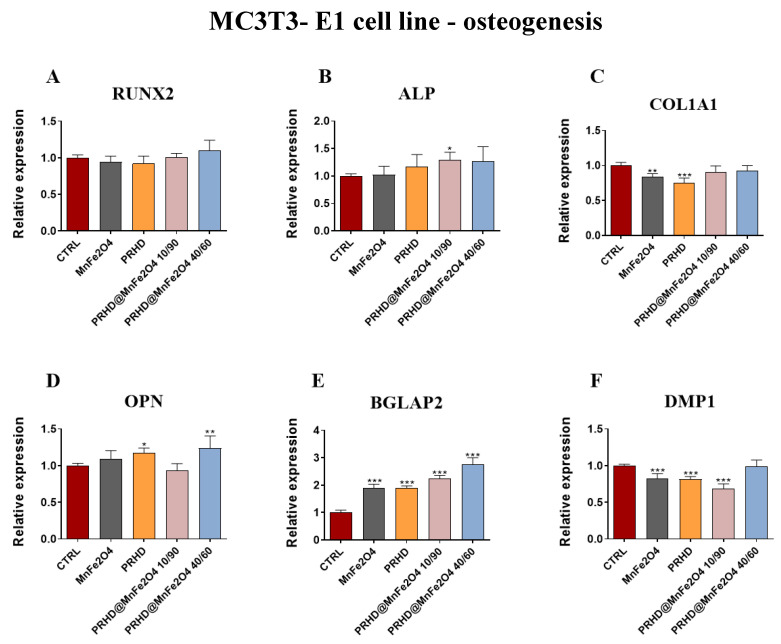
The impact of the polyrhodanine manganese ferrite binary nanohybrids and its modifications on the expression of osteogenesis associated genes: *Runx2* (**A**), *Alp* (**B**), *Col1A1* (**C**), *Opn* (**D**), *Bgalp2* (**E**), *Dmp1* (**F**) towards MC3T3-E1 cell line. Significant differences are indicated as follows: * *p* < 0.005, ** *p* < 0.001 and *** *p* < 0.001. Each experiment was performed independently three times.

In comparison, the impact of the new PRHD modifications on the osteogenic genes in the osteoblasts showed that this modification at ratio 10/90 increase the expression of *Alp* and *Bgalp2* (Figure 8B,E) while at ratio 40/60 this modification caused the increase of *Opn* and also *Bglap2* (Figure 8D,E). On the other hand, the combination of PRHD and MnFe_2_O_4_ at 10/90 ratio decreased the expression of *Dmp1* (Figure 8F). The strongest effect of the manganese ferrite binary nanohybrids, PRHD and its modifications were shown in the case of two genes, *Bglap2* and *Dmp1* (Figure 8E,F).

**Figure 9:**
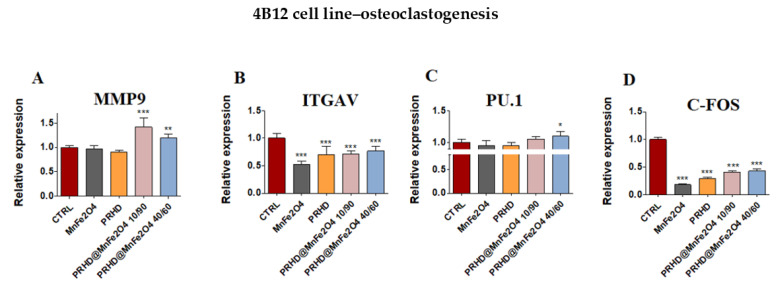
The impact of the polyrhodanine manganese ferrite binary nanohybrids and its modifications on the expression of genes associated with osteoclastogenesis: *Mmp9* (**A**), *Itgav* (**B**), *PU.1* (**C**), *c-fos* (**D**) towards 4B12 cell line. Significant differences are indicated as follows: * *p* < 0.005, ** *p* < 0.001 and *** *p* < 0.001. Each experiment was performed independently at least three times.

Moreover, the statistically significant changes in mRNA expression of markers related with osteoclasts was observed by us after treating them with both combinations of PRHD. On the one side, the PRHD@MnFe_2_O_4_ at ratio 40/60 as well as 10/90 increased the expression of matrix metalloprotein 9 (*Mmp9*) (Figure 9A), on the other, both of them decreased the expression of *Itgav* and *c-fos* (Figure 9B,D). However, only in the case of the PRHD@MnFe_2_O_4_-treated osteoclasts, we were able to observe the increased expression of PU.1 (Figure 9C).

## 4. Discussion

From many years, it is been known that rhodanine and its derivatives possess a variety of activities, such as antibacterial, antiviral, anticonvulsant, antidiabetic, antihistaminic, anticancer and hypnotic [14,15,16].

Nowadays, osteoporosis has become one of the most common bone disorders, which in the majority of cases results in bone fractures. One of the currently considered strategies for regeneration of bone fractures in osteoporosis patients is modulating the balance between osteoblasts and osteoclasts activity. Thus, consequently this should allow improvement of osteogenic differentiation by decreasing the activity of osteoclasts as well as by inducing bone formation processes. In this study we investigated both osteoblasts and osteoclasts activity culturing with polyrhodanine manganese ferrite binary nanohybrids (MnFe_2_O_4_).

In this study we demonstrated that PRHD and PRHD@MnFe_2_O_4_ at ratio 10/90 significantly improves pre-osteoblasts’ proliferative activity, while reducing osteoclasts activity in a dose dependent manner. There were no significant differences in the pre-osteoblast activity after culturing with higher concentration of MnFe_2_O_4_ (PRHD@MnFe_2_O_4_ 40/60) in relation to the control. Interestingly, PRHD alone negatively affects pre-osteoblast viability, reducing their proliferative activity (Figure 2). The observed effect could stand in good agreement with earlier findings of Zachanowicz et al., that showed the beneficial effect of PRHD@MnFe_2_O_4_ on human adipose-derived stem progenitor cells (ASCs) leading to their increased proliferative activity and reduced apoptosis [8,9]. The enhanced proliferative activity of pre-osteoblasts cultured with PRHD@MnFe_2_O_4_ was closely correlated with observed by us of a more developed cytoskeleton and mitochondrial network (Figure 2 and Figure 3).

In turn, we speculate that decreased proliferative activity of osteoclasts might be due to reduced mitochondrial activity leading to the apoptosis. Although, no existing data regards the effect of PRHD and PRHD@MnFe_2_O_4_ on pre-osteoblasts and osteoclasts activity to support our hypothesis exists, we decided to analyze a broad panel of gene expression involved in the apoptosis. We found that PRHD@MnFe2O4 at ratio 10/90 reduced expression of *p53* and *Casp9* mRNA levels, while PRHD@MnFe_2_O_4_ at ratio 40/60 did not induced the apoptosis. At the same time, we found that PRHD alone and PRHD@MnFe_2_O_4_ 10/90 promotes osteoclasts apoptosis by activation of *p21*, *p53*, *BAX* and *Casp9* expression (Figure 4 and Figure 5). Obtained data correlates with Markidis et al.’s findings, who found dose dependent cytotoxicity and apoptosis of preosteoblast, osteosarcoma cells and adipocytes after culturing in the presence of the superparamagnetic nanoparticles [17]

It was shown that increased expression of cytokines, such as tumor necrosis factor α (TNFα), interleukins IL-1, IL-6 or IL-17, were strongly associated with the excessive bone degradation mainly due to hyperactivation of osteoclasts and osteoblasts [18] (Figure 6 and Figure 7).

In this study we found that the PRHD and PRHD@MnFe_2_O_4_ at ratio 10/90 significantly reduced proinflammatory cytokines mRNA expression in both pre-osteoblasts and osteoclasts, which seems to be a promising light for their potential prevention effect, after treatment of inflammation related to osteoporosis. Moreover, recent data suggest the link between inflammation and osteoblasts activity, differentiation, fate, survival and finally, expression of master regulators of osteogenesis [18].

Finally, we found, that PRHD@MnFe_2_O_4_ at ratio 10/90 and PRHD@MnFe_2_O_4_ at ratio 40/60 promotes expression of alkaline phosphatases (ALP), osteopontin (OPN) and osteocalcin (OCL), while reducing expression of dentin matrix protein 1 (DMP-1). The recent study by Manolagas clarified that OCL plays an important role in being indispensable for the alignment of biological apatite crystallites parallel to collagen fibers, therefore playing a key role in bone strength, quality and mass—all critical in osteoporotic fractures’ bone regeneration [19].

Together with OCL, enhanced expression of ALP improves bone mineralization. Recently, Wennerg et al. showed that mineralization of osteoblasts was significantly delayed in alkaline phosphatase (TNAP) knockout mice as well as phenotypic abnormalities of infantile hypophosphatasia occurring in null mice [20]. Therefore, enhanced expression of ALP, OCL together with OPN indicate the beneficial effect of PRHD@MnFe_2_O_4_40/60 on preosteoblasts activity modulating their survival, fate and mineralization (Figure 8 and Figure 9).

Previously studies performed by Kwak et al. revealed that intra-articular injection of the rhodamine derivatives into the right knee of 12-week-old male C57BL/6J significantly reduced the pathogenesis of the osteoarthritis, which confirms the disease-modifying potential of rhodamine derivatives [21].

Moreover, based on known anti-inflammatory properties, the derivatives of the rhodanine seem to be a good solution in the case of inflammation corresponding to bone reconstruction.

## 5. Conclusions

In this study, we demonstrated that polyrhodanine manganese ferrite binary nanohybrids (PRHD@MnFe_2_O_4_) could stimulate the *ALP-OPN-OCN* axis involved in the regulation of osteogenic differentiation. At the same time, it was also included in the limitation of the osteoclasts’ activities.

In summary, we found that PRHD@MnFe_2_O_4_ could affect the bone metabolism in different ways according to the ratio of PRHD and MnFe_2_O_4_. Their impact was observed in the morphology changes, arrangement of the mitochondrial network and also in the profile of genes expression associated with the apoptosis, inflammation and bone remodeling.

We have confirmed that PRHD and its modification at ratio 10/90 clearly enhance the viability of the pre-osteoblasts. However, this combination has being involved in the strong anti-inflammatory effect demonstrated by the decrease of *Il1b, Tnfa* and *Tgfb* gene expression. Moreover, they were also involved in enhancing expression of genes associated with bone remodeling, such as osteopontin (OPN), osteocalcin (OCL) or alkaline phosphatase (ALP), that suggest that PRHD and its modification could be used as a potential agent in the treatment of osteoporosis in the future, although further studies on animals are strongly recommended to confirm the results obtained on the culture cells.

## Figures and Tables

**Table 1:** Sequences of primers used in the actual studies.

Gene	Forward (5′ → 3′)	Reverse (5′ → 3′)	Length of Amplicon
*p21*	TGTTCCACACAGGAGCAAAG	AACACGCTCCCAGACGTAGT	175
*p53*	AGTCACAGCACATGACGGAGG	GGAGTCTTCCAGTGTGATGATGG	287
*Casp9*	CCGGTGGACATTGGTTCTGG	GCCATCTCCATCAAAGCCGT	278
*Bad*	ACATTCATCAGCAGGGACGG	ATCCCTTCATCCTCCTCGGT	115
*Bax*	AGGACGCATCCACCAAGAAGC	GGTTCTGATCAGCTCGGGCA	251
*Bcl-2*	GGATCCAGGATAACGGAGGC	ATGCACCCAGAGTGATGCAG	141
*Runx2*	TCCGAAATGCCTCTGCTGTT	GCCACTTGGGGAGGATTTGT	130
*Alp*	TTCATAAGCAGGCGGGGGAG	TGAGATTCGTCCCTCGCTGG	198
*Col1A1*	CCAGCCGCAAAGAGTCTACA	CAGGTTTCCACGTCTCACCA	175
*Opn*	AGACCATGCAGAGAGCGAG	GCCCTTTCCGTTGTTGTCCT	340
*Bglap2*	CTCCTGAGAGTCTGACAAAGCCTT	GCTGTGACATCCATTACTTGC	320
*Dmp1*	CCCAGAGGCACAGGCAAATA	TCCTCCCCAATGTCCTTCTT	211
*Mmp9*	TTGCCCCTACTGGAAGGTATTAT	GAGAATCTCTGAGCAATCCTTGA	172
*Pu.1*	GAGAAGCTGATGGCTTGGAG	TTGTGCTTGGACGAGAACTG	175
*Itgav*	ACAATGTAAGCCCAGTTGTGTCT	TTTGTAAGGCCACTGGAGATTTA	236
*c-fos*	CCAGTCAAGAGCATCAGCAA	TAAGTAGTGCAGCCCGGAGT	248
*Il1b*	TGCCACCTTTTGACAGTGATG	TGATGTGCTGCTGCGAGATT	138
*Tnfa*	ACAGAAAGCATGATCCGCGA	CTTGGTGGTTTGCTACGACG	295
*iNos*	GACAAGCTGCATGTGACATC	GCTGGTAGGTTCCTGTTGTT	325
*Il6*	GAGGATACCACTCCCAACAGACC	AAGTGCATCATCGTTGTTCATACA	141
*Tgfb1*	GGAGAGCCCTGGATACCAAC	CAACCCAGGTCCTTCCTAAA	94
*Gapdh*	TGCACCACCAACTGCTTAG	GGATGCAGGGATGATGTTC	177

*p21:* cyclin-dependent kinase inhibitor 1; *p53:* tumor suppressor factor; *Casp9:* caspase 9; *Bad:* Bcl-2 associated agonist of cell death; *Bax:* Bcl-2 associated X protein; *Bcl-2:* B-cell lymphoma 2; *RUNX-2:* runt-related transcription factor 2; *Alp:* phosphatase alkaline; *Col1A1:* collagen alpha-1 chain precursor; *Opn:* osteopontin; *Bglap2:* bone-carboxyglutamic acid-containing protein; *Dmp-1:* dentin matrix protein 1; *Mmp-9:* matrix metalloproteinase 9; *PU.1:* protein in human encoded by the SPI1 gene; *Itgav:* integrin subunit alpha V; *c-fos:* protooncogene, cellular oncogene fos; *Il1b:* interleukin 1 beta; *TNFα:* tumor necrosis factor alpha; *iNOS:* inducible nitric oxide synthetase; *Il6:* interleukin 6; *Tgfb1:* transforming growth factor beta 1; *Gapdh:* glyceraldehyde-3-phosphate dehydrogenase.

## Data Availability

The data presented in this manuscript are available on request from the corresponding author.

## Abbreviations

ASCs	adipose stem progenitor cells
ATCC	American Type Cell Culture
ALP	alkaline phosphatase
ATR	attenuated total reflection
BAD	Bcl-2 associated agonist of cell death
BAX	Bcl-2 associated X protein
Bcl-2	B-cell lymphoma 2
BGLAP	bone gamma-carboxyglutamic acid-containing protein (OC)
BMD	bone mass
BMSCs	bone marrow-derived mesenchymal stem cell progenitor cells
CASP	caspase
COL1A1	collagen alpha-1 (I) chain precursor
CSCM	30% calvaria-derived stromal cell conditioned media
DEPC	sterile filter water treated with diethyl pyrocarbonate
DMP1	dentin matrix acidic phosphoprotein 1
ECM	extracellular matrix
EVs	extracellular vesicles
FTIR	Fourier transform infrared spectroscopy
FBS	fetal bovine serum
GAPDH	glyceraldehyde 3-phosphate dehydrogenase
IL	interleukin
ITGAV	integrin subunit alphaV
iNOS	inducible nitric oxide synthase
MEMα	minimum essential medium α
MMP	matrix metalloproteinase
OC	osteocalcin (BGLAP)
OPN	osteopontin
OS	osteoporosis
p21	cyclin-dependent kinase inhibitor 1
p53	tumor suppressor factor
PU.1	protein in human encoded by the SPI1 gene
PRHD	polyrhodanine
PRHD@MnFe_2_O_4_	polyrhodanine manganese ferrite binary nanohybrids
RUNX2	runt-related transcription factor 2
SEM	scanning electron microscope
TEM	transmission electron microscope
TGA	polymer layer thermogravimetric analysis
TGFβ	transforming growth factor β
TNFRSF	tumor necrosis factor receptor superfamily
TRAIL	tumor necrosis factor-related apoptosis-inducing ligand
XRD	X-ray powder diffraction technique
